# An instrument of screening criteria to assess individualized high-risk sexual behavior to be applied in the Brazilian population: translation and cross-cultural adaptation

**DOI:** 10.1016/j.htct.2024.06.009

**Published:** 2024-09-07

**Authors:** Miriane Lucindo Zucoloto, Guilherme Galdino, Edson Zangiacomi Martinez

**Affiliations:** a Fcauldade de Medicina de Ribeirão Preto, Universidade de São Paulo, Ribeirão Preto, São Paulo, Brazil; b Escola Nacional de Saúde Pública, Comprehensive Health Research Center, Universidade NOVA de Lisboa, Lisboa, Portugal

**Keywords:** Risk behavior, Blood donation, Blood safety, Equal treatment, Sexual orientation

## Abstract

**Background:**

Alternative approaches have been proposed to ensure a safe and equitable screening process for blood donation that treats all people equally, regardless of gender identity or sexual orientation. The terms ‘neutral approach’ and ‘individualized risk assessment’ have been used to describe this goal. To facilitate research and implementation of these concepts in blood donation contexts and health services in Brazil, we propose a Portuguese version of the ‘for the assessment of individualized risk screening criteria’ (FAIR) screening criteria.

**Methods:**

The FAIR screening criteria are 12 questions that assess sex, sexuality, ethnicity, and the extent to which participants engaged in each targeted sexual behavior. The aim of FAIR is to reduce error while increasing reliable and accurate reporting of sexual behaviors associated with both objective and subjective estimates of infection risk. The FAIR screening criteria were translated and cross-culturally adapted using a systematic approach with standardized procedures appropriate for adapting instruments that track behaviors.

**Results:**

A version that is appropriate for use with the Brazilian population was produced employing the following steps: expert translations, harmonization, consensus version, expert back-translation, revision, panel of experts, cognitive interviewing, and finalization.

**Conclusion:**

The Portuguese version of FAIR was proposed, and because of its straightforward, simple language and focus on specific and frequent behaviors in some populations, it has the potential to be used in a variety of contexts involving the screening of high-risk sexual behavior in Brazil.

## Introduction

When cases of AIDS began to appear in North America in the late 1970s and early 1980s, men who had sex with men (MSM) were considered a high-risk group, as were hemophiliacs and Haitians.^1^^,^^2^ This was primarily due to the disease's high prevalence in these population at a time when little was known about the virus and its transmission routes. The first case of HIV transmission through blood transfusion was recorded in 1981; in 1984 it was confirmed that it could be transmitted through this route. Screening blood donors for sexual behaviors considered high-risk became mandatory at this point.^3^^,^^4^

In 1983, the North American Food and Drug Administration (FDA) began its attempts to minimize the transmission of HIV through blood donation, developing awareness and information strategies about the virus and about risk behaviors that could expose donors to infection. However, the FDA recognized the need to intervene more directly in hemotherapy processes. As a result, the first policy prohibiting MSM from donating blood indefinitely was implemented in September 1985.^1^^,^^5^

In Brazil, the policy of excluding MSM from donating blood emerged in May 1985 with ordinance no 236 of the Ministry of Health. Even though its resolution did not explicitly include the exclusion of MSMs, it mentioned members of risk groups.^6^ Since then, there have been notable improvements in HIV treatment, although the infection still has important health consequences.^7^

Over the subsequent decades, remarkable advances in HIV testing and treatment, including the most sensitive diagnostic technology, nucleic acid amplification testing, have allowed progressive reductions in deferral periods in many countries, moving from a definitive deferral to a temporary deferral of five years, 12 months or three months. 8, 9, 10, 11, 12, 13, 14 A significant drawback of this approach is that, even for a brief period of deferral, all sexually active MSM - including those in committed monogamous relationships - are effectively barred from giving blood. It also does not identify heterosexual donors who engage in potentially risky sexual behaviors such as multiple partners or unprotected sex, which may affect the residual risk of transfusion-transmitted HIV depending on the epidemiology of transmission in a given country.^15^, ^16^, ^17^, ^18^, ^19^

Some researchers and advocates for the rights of the lesbian, gay, bisexual, transgender, intersex, queer/questioning, asexual etc. (LGBTQIA+) community have long argued that the ban of MSM is discriminatory and that different strategies for assessing high-risk behaviors already exist. Groups such as the Human Rights Campaign advocated for the US FDA to review donation eligibility in order to assess the risk of sexual behaviors equally, without regard to sexual orientation or gender identity. The UK group For the Assessment of Individualized Risk (FAIR)^20^ has conducted studies to determine whether it is possible to adopt a more individualized risk assessment approach to blood donor selection policy while also ensuring safe blood supply to patients. According to the group, there is no single way to ensure the safety of the blood supply. Nonetheless, the literature generally agrees that the two primary steps required to guarantee safety are (a) serological testing of all potential donors and (b) precise identification of safe donors through behavioral and clinical screening.^21^^,^^22^

The findings of the studies conducted by the FAIR group recommend several adjustments to guarantee a safe and equitable screening process that treats all individuals equally, irrespective of their gender identity or sexual orientation. These adjustments have been dubbed as a ‘neutral approach’ or ‘individualized risk assessment’.^20^ The advantage of this sex-neutral approach is that it does not stigmatize gay men by classifying all sexually active MSM as high-risk. However, regardless of the sexual orientation of donor candidates, this requires asking all donors a new set of personal questions and personalizing the pre-donation clinical interview by addressing high-risk behaviors that may affect transfusion risk.^20^, ^21^, ^22^, ^23^

In Brazil, the rules that temporarily excluded MSM from blood donation were changed by a decision of the Supreme Court in 2020. The Supreme Court deemed that the regulations of the National Health Surveillance Agency (Anvisa) and the veto of the act by the Ministry of Health were discriminatory.^6^ Consequently, a project in the Brazilian house of Senate that called for the outlawing of discrimination against blood donors based on sexual orientation was approved at the end of 2021. Nevertheless, no novel methods of behavioral screening have been introduced despite modifications. Moreover, no research has been done to restructure the behavioral and clinical screening interview and suggest a neutral strategy that takes transfusion risk into account, in contrast to other nations like the United Kingdom and Canada.^21^^,^^23^, ^24^, ^25^

Thus, this study aims to propose a Portuguese version of the FAIR screening criteria that has been adapted for use in studies on the implementation of a neutral approach based on individual risk in healthcare services in Brazil.

## Methods

### For the assessment of individualized risk screening criteria

The screening criteria proposed in the FAIR Steering group^20^ sought to identify sexual behavior questions that minimize error while increasing reliable and accurate reporting of sexual behavior associated with both objective and subjective estimates of infection risk. The questionnaire consists of 12 items with Yes or No responses that assess sex, sexuality, ethnicity, and the extent to which participants engage in each targeted sexual behavior/relationship. Three additional questions were included in the assessment of the psychometric properties of the questionnaire to determine the extent to which the participants believed they could accurately recall their behavior, whether they thought the question was inappropriate to ask, and whether being asked the question would deter them from donating blood. These additional questions were also part of the translation and cross-cultural adaptation process of the present study, aiming to propose a comprehensive instrument to support the subsequent stages of validation and implementation in different Brazilian populations.

### Translation and cross-cultural adaptation steps

The FAIR screening criteria were translated and cross-culturally adapted using a systematic approach, with standardized procedures chosen from the literature as the most appropriate for adapting instruments for tracking behaviors,^26^, ^27^, ^28^ as well as the World Health Organization guidelines for adapting instruments.^29^ The translation process involved three major steps:

1) Expert translations: three Portuguese-native bilingual translators with a background in text translation and revision, each independently produced a Portuguese version of the items.
2) Harmonization and consensus version: Two researchers with experience in translating studies and cross-cultural adaptation of scales, furnished with the three translated versions, proposed a consensus version of the instrument that included the best-translated option for each item while taking into account the instrument's suitability for the target population and study objectives.
3) Expert back-translation: This consensus version was then back-translated by an English-native bilingual professional with prior experience in translating and revising texts. To participate in this stage, the professional had no knowledge of or access to the original version of the items.
4) Revision and final version: At the end of the process, the two researchers responsible for the consensus version reviewed the back-translation and concluded the instrument translation stage by proposing the final Portuguese version of each item to proceed to the next stages of cross-cultural adaptation.

The cross-cultural adaptation process entailed adapting, transforming, or confirming that the items in the consensus version were appropriately adapted for the study's target population. A systematic approach was used to accomplish this based on the proposal of Wild et al.^27^ and Guillemin et al.,^26^ which involves three main steps:

1) Panel of Experts: This step was critical to determine whether the translated items could be improved to meet the study objectives, in addition to confirming the semantic, idiomatic, cultural, and conceptual equivalences. Semantic equivalence refers to the meaning of words in terms of vocabulary and grammar; idiomatic equivalence refers to the equivalence of expressions and meanings in different languages; cultural equivalence refers to the adaptation of the context to the study target audience; and conceptual equivalence refers to the preservation of the instrument's original concept. Because it is a practical questionnaire for tracking high-risk sexual behaviors that may influence transfusion risk, five health professionals with experience in human behavior, risk behavior, or directly linked to the blood donation process participated in this step. The experts were asked by e-mail to classify each questionnaire item based on the equivalence classifications as: ‘non-equivalent sentence’, ‘impossible to assess the equivalence of the sentence without reviewing it’, ‘equivalent sentence, but requiring minor revision’ or ‘totally equivalent sentence’. In these circumstances, suggestions for revisions and simplifications of items were requested. The content validity index (CVI) was calculated for each item considering the proportion of notes as non-equivalent - with a CVI of 0.70 or less indicating the need to return the instrument to the initial stage.^30^
2) Cognitive interviewing: Based on guidelines given by Willis,^31^ this step was conducted with a small group of participants who are members of the target population. Cognitive interviewing was used to test comprehension, interpretation, and the cultural relevance of the translation. Researchers used their social networks to find volunteers for this step of the study, therefore using a convenience sample. The invitation to participate was sent via e-mail or social media chat, and participants were chosen in order of interest. Fifteen people who recognized themselves as MSM were selected to participate. The interviews were carried out by a single researcher.
  The Google Meet platform was used for cognitive interviewing, allowing both the participant and the interviewer to communicate and establish a dialogue. The Portuguese version of the FAIR screening criteria was hosted on RedCap, a platform used to create web surveys.^32^ Some sociodemographic questions (age, educational level, monthly income, and gender identity) were included in the online survey to characterize the participants.
  All interviews was conducted independently at a scheduled time. After a presentation explaining the objectives of that stage of the study and how their participation would be conducted, a link containing the questionnaire to be completed was shared with the participant. The interviewer remained available while the participants were answering the questions in case of any doubts or questions. At the conclusion of the questionnaire, the interviewer revised the items with the participants and asked them about their comprehension of the instrument, and their responses were noted.
  Following this, another web survey link was sent that contained the following questions:
  “How uncomfortable did you feel while answering the questions of the previous questionnaire?”;
  “In case you felt very uncomfortable, would you please describe in a few words the reason for the discomfort?”;
  “Do you consider any of the items/words used in the previous questionnaire stigmatizing or inappropriate?”;
  “If you consider any of the items/words used in the previous questionnaire stigmatizing or inappropriate, would you write down what we could change, please?”;
  “Would you change anything in this questionnaire and, if so, what would you change?”;
  “In your opinion, what is this questionnaire for?”
3) Cognitive interviewing results review and finalization: The final review of the instrument, considering the results of the previous stages, was carried out by the researchers, and the final version of the instrument was proposed.

### Ethical aspects

The Ethics Committee for Research with Human Beings of the Hospital das Clínicas de Ribeirão Preto approved this project and the associated terms of free and informed consent (CAAE: 71,364,123.0.0000.5440). Informed consent was obtained from all those who agreed to participate in the research and was presented on the first page of the electronic questionnaire in the cognitive interview and expert panel stages. Consent was given when participants clicked on the “I accept the terms of participation” button of the online forms.

No personal information (such as name, personal identification number, address) was collected or stored. The IP address was not captured by REDCap and the electronic questionnaire did not use cookies to collect personally identifiable information about respondents.

## Results

The original version of the FAIR screening criteria was translated and went through all planned stages of cross-cultural adaptation until the proposal of a final version suitable for its application in the Brazilian population. A better description of some steps are provided in the results section so that the process can be better understood.

The cross-cultural adaptation stages began with an expert panel. This stage was attended by five professionals: two specialists in adapting scales on human behavior, a nurse involved in the blood donation process, and two researchers in the area of sexual risk behaviors of vulnerable populations. All experts (nurse and researchers) had previous experience with scale adaptation. As a result, all items were categorized as ‘totally equivalent sentences’ based on the expert's evaluation of the equivalency of the translated items with no additional major modifications or reviews being required. Only a few suggestions for capitalizing or highlighting certain words were made by the experts which the authors deemed relevant. These changes were made to the final version of the instrument.

Data for the cognitive interviewing step were collected between November 8th and November 25th 2023. A brief characterization of the participants in this stage was obtained. The sample was composed of 15 MSM individuals with a mean age of 23.4 (standard deviation: 2.7) years. Among the participants, 14 classified themselves as men and one as non-binary/queer. Most have incomplete higher education. In terms of monthly income, the majority of participants reported earning between two and four minimum wages, with one participant reporting less than one minimum wage.

The duration of each interview session was from 10 to 15 min. As a result, all participants classified the items as easy to understand and had no doubts regarding the content. One participant admitted discomfort while answering certain items. When asked about the source of his discomfort, the participant stated, as an example, that he felt guilty for not using condoms in his sexual relationships. No other respondent reported feeling uncomfortable while filling out the questionnaire. Additionally, none of the 15 participants considered any of the items or words used in the items of the Portuguese version of the FAIR screening criteria as stigmatizing or inappropriate, nor would they change anything in the questionnaire. In response to the question “What is this questionnaire for?”, ten participants believed it was applied for screening behaviors, sexual health, and sexually transmitted infections (STIs); four believed it was an instrument for collecting data for scientific studies; and one declared he did not know.

After reviewing all of the steps and ensuring that they were completed, the researchers finalized the version of the instrument, as presented in Table 1 along with its original version for comparison.

**Table 1:** Original and Portuguese final version of the For the Assessment of Individualized Risk screening criteria (FAIR).

Item	Original version of the FAIR screening criteria*	Portuguese version of the FAIR screening criteria
1	Do you believe your current relationship is exclusive (neither of you have sex with another people)?	Você acredita que seu relacionamento atual seja exclusivo (nem você nem seu parceiro tem tido relações sexuais com outras pessoas)?
2	How many sexual partners have you had in the last 3 months, including oral, anal or vaginal sex? (please indicate the number)	Quantos parceiros sexuais você teve nos últimos 3 meses, incluindo sexo oral, anal ou vaginal? (Por favor indique o número)
3	Have you had any new sexual partners in the last 3 months?	Você teve algum novo parceiro sexual nos últimos 3 meses?
4	Have you had ONLY oral sex in the last 3 months?	Você fez APENAS sexo oral nos últimos 3 meses?
5	Have you had anal sex in the last 3 months?	Você fez sexo anal nos últimos 3 meses?
6	Did you use any drugs (excluding Viagra or cannabis) before or during sex to improve your sexual experience in the last 3 months?	Você usou alguma droga (que não seja Viagra ou maconha) antes ou durante o sexo para melhorar suas experiências sexuais nos últimos três meses?
7	Did you use condoms every time you had sex (including oral, anal or vaginal sex) in the last 3 months?	Você usou preservativos TODAS as vezes que teve relações sexuais (incluindo sexo oral, anal ou vaginal) nos últimos 3 meses?
8	Have you given penetrative sex in the last 3 months?	Você fez sexo com penetração nos últimos 3 meses, sendo você a pessoa que penetrou?
9	Have you received penetrative sex in the last 3 months?	Você recebeu penetração sexual nos últimos três meses?
10	Have you been diagnosed with or received treatment for gonorrhea, syphillis or chlamydia in the past 3 months?	Você foi diagnosticado ou fez tratamento para gonorreia, sífilis ou clamídia nos últimos 3 meses?
11	Have you taken PEP in the last 3 months (PEP is a treatment that can stop HIV infection after the virus has entered a person's body. It must be taken within 72 h of exposure)?	Você tomou PEP nos últimos 3 meses? (PEP é um tratamento que pode interromper a infecção pelo HIV depois que o vírus entra no corpo de uma pessoa. Deve ser tomado até 72 horas **após a exposição**)
12	Have you taken PREP in the last 3 months (PREP is a drug taken by people before sex that reduces the risk or getting HIV)?	Você tomou PREP nos últimos três meses? (PREP é um medicamento tomado pela pessoa **antes da relação** sexual para reduzir o risco de contrair HIV)
Additional questions	How accurate is your answer? (Complete guess, Pretty accurate, Completely accurate)	O quão precisa/exata foi sua resposta para esta pergunta? (Sem nenhuma precisão / Pouco precisa/ Muito precisa)
	Is this question inappropriate to ask? Yes/ No	Essa é uma pergunta inapropriada para se fazer? (Sim/Não)
	Would being asked this question put you off donating blood? (Yes/ Quite Likely/ Not very likely/ No/ Not sure)	Se essa pergunta fosse feita, ela te desencorajaria a doar sangue? (Sim/ Muito provavelmente / Pouco provavelmente/ Não / Não tenho Certeza)

⁎ FAIR Group 2020.

## Discussion

After all the steps as presented in detail, the present study suggests that the translated and adapted version of the FAIR screening criteria is suitable for application in studies on high-risk sexual behavior in the Brazilian population. As previously stated, the FAIR screening criteria were designed as a neutral approach in the context of clinical screening for blood donation, aiming to reduce transfusion risk.^20^ However, due to its straightforward, simple language and focus on specific and frequent behaviors in some populations, this instrument may also be helpful both for scientific studies and clinical practice in different contexts in the public health area.

In the official publication of the FAIR steering group's conclusions,^20^ the authors proposed two versions of the instrument, which were used in different contexts/surveys, namely 1) Reports of individual actual behavior, considering the general population, and 2) Perceptions of normative behavior, considering the population of blood donors. The two versions differ in minor aspects, with the main distinction being the separation of questions about the use of PREP and PEP. Two questions were used in the version intended for the donor population, one for PREP and the other for PEP, yielding a total of 12 items. For the general population, the authors combined these questions into a single question (“In the last three months, have you taken PrEP or PEP?”), yielding an instrument with 11 items. In the current study, we propose an adapted version that addresses PrEP and PEP separately, as this is a more complete version of the instrument. In this regard, we recommend that adaptation needs and the possibility of combining items be evaluated before applying it in subsequent studies, considering its objectives and its contexts of use.

The applied methodology was designed to avoid language that is not inclusive or stigmatizes some population groups. Therefore, no question was recognized as such, a result of the cognitive interviewing stage. Nonetheless, it is well known that certain inquiries concerning sexual behavior frequently cause discomfort, particularly in medical settings.^22^^,^^33^

A study conducted by Haw et al.^22^ sought to identify strategies to reduce discomfort in answering specific questions about high-risk sexual behavior in blood donation candidates. Several strategies to lessen discomfort were mentioned by participants, both explicitly in their suggestions and implicitly in their discussions about how comfortable they felt answering the presented questions. According to the findings, reducing discomfort may involve providing explanations that go into varying degrees of detail regarding the necessity and purpose of the questions. Participants also suggested that more specific questions would lessen guesswork and that ambiguity in the use of spoken or written language may lead to uncertainty. Finally, it was preferable to answer questions in a self-administered questionnaire, such as a web-based app, rather than in a face-to-face interview.

Still considering the interrogatory interview step, one participant expressed a sense of guilt while filling out the FAIR instrument. According to Thomas et al.,^34^ the MSM population is more susceptible to internalized homophobia and low self-esteem, which can lead to increased risk-taking and poor decision-making. Given that accessibility in healthcare includes proper language use,^35^ extra efforts are required to prepare professionals involved in screening processes or medical interviews so that they do not stigmatize the patients/blood donation candidates, thereby avoiding discomfort and feelings of guilt.

As a conclusion, the Portuguese version of the FAIR screening criteria for high-risk sexual behavior screening is available for use in future studies or for implementation in healthcare services. Guidance and information about the instrument's content should be tailored to the unique circumstances of each application, taking into account the discomfort that different population groups may experience. If a personal interview is the preferred method of application, team training and additional employee preparation may be required.

## Funding sources

This work was supported by the 10.13039/501100001807São Paulo Research Foundation [Grants: #2023/10473-5; #2020/02187-4].

## Conflicts of interest

The author declares no conflicts of interest.
